# Multicenter machine learning prediction of postoperative heart failure in elderly patients highlighting inflammatory markers, metabolic comorbidities, and perioperative tachycardia

**DOI:** 10.3389/fmed.2026.1833776

**Published:** 2026-04-29

**Authors:** Wenyi Du, Feng Zhan, Wentan Chen, Miao Zhang, Chengyu Shi, Yun Zhang, Chao Jiang, Yu Zhang

**Affiliations:** 1 Department of Hepatobiliary Surgery, Yixing People's Hospital Affiliated to Jiangsu University, Yixing, China; 2 General Surgery Centre, Yixing People's Hospital Affiliated to Jiangsu University, Yixing, China

**Keywords:** external validation, heart failure, inflammatory response, machine learning, predictive model

## Abstract

**Background:**

Postoperative heart failure (HF) represents a prevalent and serious complication among elderly surgical patients, markedly increasing perioperative morbidity, prolonging hospitalization, and elevating mortality risk. Early identification of high-risk individuals is therefore of substantial clinical importance for optimizing perioperative management. Conventional statistical models are inherently limited in capturing complex, nonlinear interactions among variables, whereas machine learning (ML) approaches offer distinct advantages in predictive performance and individualized risk stratification.

**Methods:**

In this retrospective multicenter study, 1,562 elderly patients were consecutively enrolled from six hospitals, comprising 757 individuals in the internal cohort and 805 in the external validation cohort. Independent risk factors for postoperative HF were identified through univariate and multivariate logistic regression analyses. Five machine learning algorithms—extreme gradient boosting (XGBoost), random forest (RF), support vector machine (SVM), k-nearest neighbours (KNN), and multilayer perceptron (MLP)—were applied to rank feature importance. Variables consistently identified by both statistical and machine learning approaches were subsequently integrated into model development. The generalizability of the internal model was assessed using tenfold cross-validation. Model performance was comprehensively evaluated using receiver operating characteristic (ROC) curves, area under the curve (AUC), calibration plots, decision curve analysis (DCA), learning curves, Kolmogorov–Smirnov (KS) statistics, and confusion matrices. Model interpretability was further interrogated using SHapley Additive exPlanations (SHAP).

**Results:**

Postoperative HF occurred in 66 patients (8.72%) within the internal cohort. Multivariate analysis and ML-based feature selection consistently identified sex, body mass index (BMI), diabetes mellitus, hypertension, hyperlipidaemia, history of malignancy, intraoperative tachycardia, and postoperative inflammatory markers (neutrophil-to-lymphocyte ratio (NLR) and C-reactive protein (CRP)) as key predictors. Among the models, XGBoost demonstrated superior performance, achieving an AUC of 0.979 in the training set, 0.937 in the internal validation set, and 0.92 in the external validation cohort. Tenfold cross-validation further confirmed robust generalizability (AUC = 0.933; accuracy = 0.908). SHAP analysis indicated that postoperative NLR and CRP made substantial contributions to model predictions, while individual-level SHAP visualizations further delineated the specific contributions of each variable to patient-level risk estimation.

**Conclusion:**

We developed and externally validated a machine learning–based predictive model for postoperative HF in elderly patients. The XGBoost model exhibited excellent discrimination, robust calibration, and promising clinical utility. SHAP-based interpretability analyses highlighted the pivotal contributions of inflammatory markers, metabolic comorbidities, and perioperative tachycardia, providing a reliable tool for individualized perioperative risk assessment in the elderly population.

## Introduction

With the accelerating pace of global population aging, the number of elderly patients undergoing surgical procedures continues to rise. This population is frequently burdened by multiple chronic comorbidities, including hypertension, coronary artery disease, diabetes mellitus, and chronic kidney dysfunction, accompanied by a diminished physiological reserve and reduced tolerance to surgical stress (1, 2). Concurrently, the aging cardiovascular system undergoes structural and functional alterations—such as reduced left ventricular compliance, myocardial hypertrophy, and impaired cardiac output—which collectively predispose elderly patients to haemodynamic instability and cardiac dysfunction in the perioperative setting (3–6). The coexistence of polypharmacy and complex comorbidity profiles further complicates preoperative evaluation and perioperative management. Consequently, elderly patients not only face heightened intraoperative risk but also exhibit a substantially increased incidence of postoperative complications, among which cardiovascular events are the most prevalent.

Among these, postoperative heart failure (HF) is recognized as one of the most severe complications (7, 8). HF is a complex clinical syndrome arising from structural or functional cardiac abnormalities that impair ventricular filling or ejection, clinically manifesting as dyspnoea, fatigue, and fluid retention (9, 10). Within the perioperative context, surgical trauma, anesthesia-related effects, fluctuations in volume status, and systemic inflammatory responses may collectively precipitate or exacerbate cardiac dysfunction, ultimately leading to postoperative heart failure. In elderly patients, diminished cardiovascular reserve and the presence of multiple underlying conditions further compromise their capacity to withstand perioperative stress, rendering them particularly susceptible to heart failure–related complications. Once established, postoperative heart failure is associated with a marked increase in perioperative mortality, prolonged mechanical ventilation, greater demand for intensive care, and extended hospital stays, thereby imposing a substantial burden on both patient outcomes and healthcare systems. Early identification of individuals at high risk is therefore of critical importance for optimizing perioperative management strategies and improving clinical outcomes.

Previous studies have demonstrated that the development of postoperative heart failure is influenced by a constellation of factors, including age-related alterations in myocardial structure and function, pre-existing cardiovascular disease, metabolic disturbances, and perioperative haemodynamic fluctuations (11–14). However, conventional risk stratification models, which predominantly rely on regression-based statistical approaches, are inherently limited in their ability to accommodate high-dimensional data and complex nonlinear relationships (15–18). Moreover, many existing studies are derived from single-center cohorts or relatively small sample sizes, lacking robust multicenter real-world data and rigorous external validation; consequently, the generalizability of these predictive models across diverse clinical settings remains uncertain (19–21).

In recent years, machine learning (ML) approaches have emerged as powerful tools for clinical risk prediction. Compared with traditional statistical models such as logistic regression, ML algorithms offer several distinct advantages. They are capable of handling high-dimensional, heterogeneous datasets and can automatically uncover latent patterns and nonlinear associations without requiring prespecified assumptions (22). Furthermore, ML methods excel in capturing complex interactions among variables, thereby providing a more faithful representation of multifactorial clinical processes. Many algorithms, including random forest and extreme gradient boosting (XGBoost), exhibit strong robustness to missing data and outliers, enabling stable performance in real-world clinical datasets. Importantly, through feature importance ranking and model interpretability techniques—such as SHapley Additive exPlanations (SHAP)—ML models not only achieve high predictive accuracy but also provide quantitative insights into the contributions of key clinical variables, thereby enhancing interpretability and clinical credibility (23, 24). In addition, ML models demonstrate strong transferability and generalizability across multicenter datasets, and their performance can be rigorously evaluated using cross-validation and external validation strategies, supporting their applicability across diverse patient populations and clinical environments.

Despite these advantages, multicenter ML-based studies focusing on the prediction of postoperative heart failure in elderly patients remain scarce. In particular, substantial gaps persist in terms of model interpretability, cross-center generalizability, and clinical applicability, which limit the translation of existing models into routine practice. To address these challenges, the present study leverages a multicenter retrospective cohort to integrate clinical characteristics and perioperative variables, with the aim of developing and validating a machine learning–based predictive model for postoperative heart failure in elderly patients. The model's generalizability is further assessed using an independent external cohort, while SHAP-based analyses are employed to enhance interpretability, thereby providing a robust and clinically meaningful tool for perioperative risk stratification and individualized patient management.

## Materials and methods

### Study population selection

The data utilized in this study were retrospectively collected from the clinical databases of six hospitals: Wuxi People's Hospital Affiliated to Nanjing Medical University, Wuxi Second People's Hospital, Yixing People's Hospital, Tengzhou Central People's Hospital, Gaomi People's Hospital, and Tengzhou Hospital of Traditional Chinese Medicine, spanning the period from January 2020 to January 2025. Inclusion criteria were as follows: (1) age ≥65 years; (2) patients undergoing elective or emergency surgery within one of the following five systems during hospitalization: digestive system (including but not limited to gastrectomy, radical colorectal resection, partial hepatectomy, and pancreaticoduodenectomy); orthopaedics (including but not limited to total hip/knee arthroplasty, spinal internal fixation, and femoral neck fracture fixation); urinary system (including but not limited to radical nephrectomy, radical cystectomy, and transurethral resection of the prostate); thyroid/breast surgery (including but not limited to radical or total thyroidectomy and radical or modified radical mastectomy); and gynaecology (including but not limited to total abdominal hysterectomy and cytoreductive surgery for ovarian cancer); and (3) new-onset postoperative heart failure occurring within 30 days after surgery or during the same hospitalization.

Exclusion criteria included: (1) pre-existing diagnosis of heart failure (any ejection fraction type) or a history of heart failure; (2) severe structural heart disease, such as significant valvular disease requiring intervention, obstructive hypertrophic cardiomyopathy, or complex congenital heart disease; (3) severe preoperative hepatic or renal dysfunction; (4) any serious comorbidity expected to limit life expectancy; (5) acute myocardial infarction, unstable angina, or cerebrovascular accident within 1–3 months prior to surgery; (6) multiple surgical procedures during the same hospitalization; (7) cardiac and major vascular surgery, cranial surgery, or maxillofacial/oral surgical procedures during hospitalization; (8) abnormal BNP, NT-proBNP, or cardiac troponin I/T results within 48 h preoperatively; and (9) significant missing key clinical data (e.g., preoperative echocardiography or postoperative critical biomarker measurements) that would compromise statistical analyses.

This retrospective study was approved by the Ethics Committees of Wuxi People's Hospital Affiliated to Nanjing Medical University, Wuxi Second People's Hospital, Yixing People's Hospital, Tengzhou Central People's Hospital, Gaomi People's Hospital, and Tengzhou Hospital of Traditional Chinese Medicine. Written informed consent was obtained from all patients, and personal information was anonymized. The study was conducted under ethical approval numbers KY22086 and IEC-AF/17-1.1.

### Collection of relevant study data

In this study, relevant clinical variables were systematically collected across distinct perioperative time points, encompassing preoperative, intraoperative, and postoperative phases. Preoperative variables, obtained within 24 h prior to surgery, included demographic characteristics (sex, age, body mass index [BMI], smoking history, and alcohol consumption), baseline clinical assessments (American Society of Anesthesiologists [ASA] score, Nutritional Risk Screening 2002 [NRS2002] score), past medical history (anemia, hypothyroidism, hyperlipidaemia, hypertension, diabetes mellitus, and chronic obstructive pulmonary disease), and previous surgical history. Preoperative laboratory indices, including serum albumin (ALB), were also recorded.

Intraoperative variables encompassed anaesthetic modality, surgical approach, urgency of the procedure, operative duration, intraoperative blood loss, episodes of intraoperative hypotension, intraoperative tachycardia, intraoperative oxygen saturation (SpO_2_), and intraoperative transfusion requirements.

Postoperative variables were collected within 48 h after surgery and included postoperative complications and laboratory parameters, such as hypotension, infection, and inflammatory markers (procalcitonin [PCT], C-reactive protein [CRP], and serum amyloid A [SAA]). Additionally, postoperative laboratory assessments included the neutrophil-to-lymphocyte ratio (NLR), serum creatinine (SCr), blood urea nitrogen (BUN), and urinalysis parameters (proteinuria and hematuria).

The prediction starting point of this model was defined as the immediate postoperative period (0 h), when the patient leaves the operating room and is transferred to the intensive care unit or general ward.

### Diagnosis of postoperative HF and determination of associated factors

This study adhered strictly to the heart failure management guidelines issued by the American College of Cardiology/American Heart Association and the European Society of Cardiology to standardize the definition of heart failure events occurring during follow-up (25–27). Patients were followed for 1 year, and upon the emergence of typical heart failure symptoms (such as dyspnoea, orthopnoea, or fatigue) and/or signs (including peripheral edema, pulmonary rales, or jugular venous distension), a comprehensive clinical assessment was initiated. All patients underwent transthoracic echocardiography using the PHILIPS Affiniti 70W system to measure left ventricular ejection fraction (LVEF).

Based on these clinical and echocardiographic evaluations, heart failure was defined as follows: patients with LVEF ≤ 40% were diagnosed with heart failure with reduced ejection fraction (HFrEF). For patients with LVEF ≥50% but with high clinical suspicion, diagnosis required corroborating objective evidence of structural or functional cardiac abnormalities, fulfilling at least one of the following criteria: (1) structural changes such as left atrial enlargement (left atrial diameter >34 mm) or left ventricular hypertrophy; (2) echocardiographic evidence of left ventricular diastolic dysfunction, primarily indicated by a mitral E/e′ ratio ≥13; (3) non-acute-phase serum N-terminal pro–B-type natriuretic peptide (NT-proBNP) levels >125 pg/mL. Using these comprehensive criteria, both HFrEF and heart failure with preserved ejection fraction (HFpEF) were included in the final heart failure analysis.

Intraoperative hypotension was defined as a mean arterial pressure (MAP) < 65 mmHg or a ≥20% decrease from baseline. Intraoperative tachycardia was defined as a heart rate >100 beats per minute or a ≥20% increase from baseline. Intraoperative hypoxemia was defined as an SpO_2_ < 90% sustained for at least 1 min.

### Missing data handling report

In this study, variables with a missing rate of less than 5% were defined as low-missingness variables, those with a missing rate between 5 and 30% were classified as moderate-to-high missingness variables, and variables with a missing rate exceeding 30% were excluded or treated as “missing” indicator variables.

Two complementary strategies were employed to address missing data according to the degree of missingness. For variables with low missingness, simple imputation was applied; categorical variables were imputed using the mode (most frequent category).

For variables with moderate-to-high missingness, multiple imputation was adopted. Taking binary variables as an example, the procedure was as follows: first, a logistic regression model was fitted using complete cases to estimate the predicted probability (*p*) that the target variable equals one for each missing observation. Subsequently, a probability-based stochastic imputation approach was implemented, whereby a random number (*u*) was drawn from a uniform distribution; if *p* ≥ *u*, the missing value was imputed as 1, otherwise as 0. By incorporating stochastic variation, this approach appropriately reflects the uncertainty inherent in the imputation process and avoids underestimation of standard errors. For multinomial variables, a similar procedure was conducted using multinomial logistic regression models. All imputation procedures were performed independently after the training–validation split to prevent information leakage.

### Development and evaluation of predictive models for machine learning algorithms

This study employed a multicenter retrospective cohort design, collecting data from elderly patients across six hospitals in two geographic regions. Following the application of predefined inclusion and exclusion criteria, patients were divided into an internal dataset and an external validation dataset. Specifically, the internal dataset comprised patients from three tertiary hospitals in the Wuxi region—Wuxi People's Hospital Affiliated to Nanjing Medical University, Wuxi Second People's Hospital, and Yixing People's Hospital—and was utilized for machine learning model development, feature selection, and hyperparameter tuning. The external dataset included patients from three hospitals in the Lunan region—Tengzhou Central People's Hospital, Gaomi People's Hospital, and Tengzhou Hospital of Traditional Chinese Medicine—and was used to evaluate model generalizability and stability, thereby testing applicability across distinct medical centers.

(1) Feature selection and preliminary statistical analysis: to identify potential predictors of postoperative HF and provide input features for subsequent machine learning modeling, traditional statistical analyses were first conducted on the internal dataset. Patients were stratified into HF and non-HF groups according to postoperative outcomes, and baseline characteristics, clinical features, and laboratory indices were compared between the groups. Continuous variables were first assessed for normality; normally distributed variables were expressed as mean ± standard deviation and compared using independent-sample *t*-tests, while non-normally distributed variables were presented as medians with interquartile ranges and compared using the Mann–Whitney *U*-test. Categorical variables were reported as counts and percentages and compared using the chi-square test or Fisher's exact test as appropriate. Variables with *P* < 0.05 in univariate analyses, together with clinically relevant variables considered strongly associated with postoperative HF, were entered into multivariate binary logistic regression to identify independent predictors, with odds ratios and 95% confidence intervals calculated.

Subsequently, five machine learning algorithms—extreme gradient boosting (XGBoost), random forest (RF), support vector machine (SVM), k-nearest neighbours (KNN), and multilayer perceptron (MLP)—were applied to the internal dataset to rank feature importance. To select core clinical predictors that were both statistically significant and stably predictive, only variables ranked among the top ten features across all five machine learning algorithms and identified as independent predictors in univariate and multivariate logistic regression were retained. This dual selection strategy ensured that the final set of core features preserved the independent risk factors identified through traditional regression analysis while integrating the ability of machine learning algorithms to capture complex nonlinear relationships, achieving a balanced trade-off between clinical interpretability and predictive stability.

(2) Upon model development, we systematically evaluated the machine learning model's performance across five dimensions: discrimination, calibration, clinical utility, internal generalization, and external transportability. Discrimination was assessed using ROC curves and AUC, with values approaching 1.0 indicating stronger discriminatory ability. Calibration was evaluated using calibration plots supplemented by Brier scores, where lower scores indicate higher predictive accuracy and stability. Clinical utility was assessed using DCA to quantify net benefit compared with “treat-all” and “treat-none” strategies.

Additionally, to compare the performance between the traditional logistic regression model and the machine learning model, we incorporated the final selected clinical indicators into a traditional logistic regression framework and constructed ROC curves, calibration curves, and DCA curves.

Internal generalization ability was assessed using multiple methods to evaluate model stability and learning capacity within the internal dataset. Tenfold cross-validation was conducted by randomly partitioning the internal dataset into ten approximately equal subsets, sequentially using one subset for validation and the remaining nine for training, repeated ten times so that each subset served once as a validation set. Averaging results across folds mitigated the influence of random data partitioning, providing an objective assessment of model stability and generalizability. Learning curves were plotted to analyze performance trends on training and validation sets as a function of increasing sample size, detecting potential overfitting or underfitting. Kolmogorov–Smirnov (KS) curves were used to evaluate the model's ability to distinguish between positive and negative outcomes, with higher KS values indicating stronger discriminatory power. Confusion matrices for both training and validation sets were constructed to display classification distributions—including true positives, false positives, true negatives, and false negatives—further assessing stability and consistency of predictions.

External transportability was evaluated by applying the internally trained and optimized model to the external dataset from the three Lunan region hospitals. Model performance in this independent cohort was assessed using ROC curves and AUC for discrimination, calibration plots for predictive accuracy, and DCA for clinical net benefit, thereby evaluating model applicability, stability, and generalizability across different geographic regions, healthcare centers, and patient populations. This multidimensional evaluation framework provided a rigorous assessment of predictive performance, stability, and clinical utility, establishing a reliable tool for early risk identification of postoperative HF in elderly surgical patients.

(3) Model interpretability using SHAP: to enhance model interpretability and quantify the contribution of each predictor to model outputs, SHapley Additive exPlanations (SHAP) were employed. SHAP, based on the Shapley value concept from cooperative game theory, decomposes the prediction of complex machine learning models into the additive contributions of each feature. In this study, SHAP summary plots were first generated to rank the global importance of all variables included in the model, evaluating the overall contribution of each clinical feature to postoperative HF risk prediction. SHAP dependence plots were subsequently used to examine the relationship between key variables and predicted risk, illustrating how changes in feature values influence model output. Finally, SHAP decision plots provided patient-level explanations, demonstrating how individual clinical variables collectively contribute to the predicted outcome for each patient.

## Results

### Baseline clinical characteristics and identification of risk factors for postoperative HF

**Figure 1:**
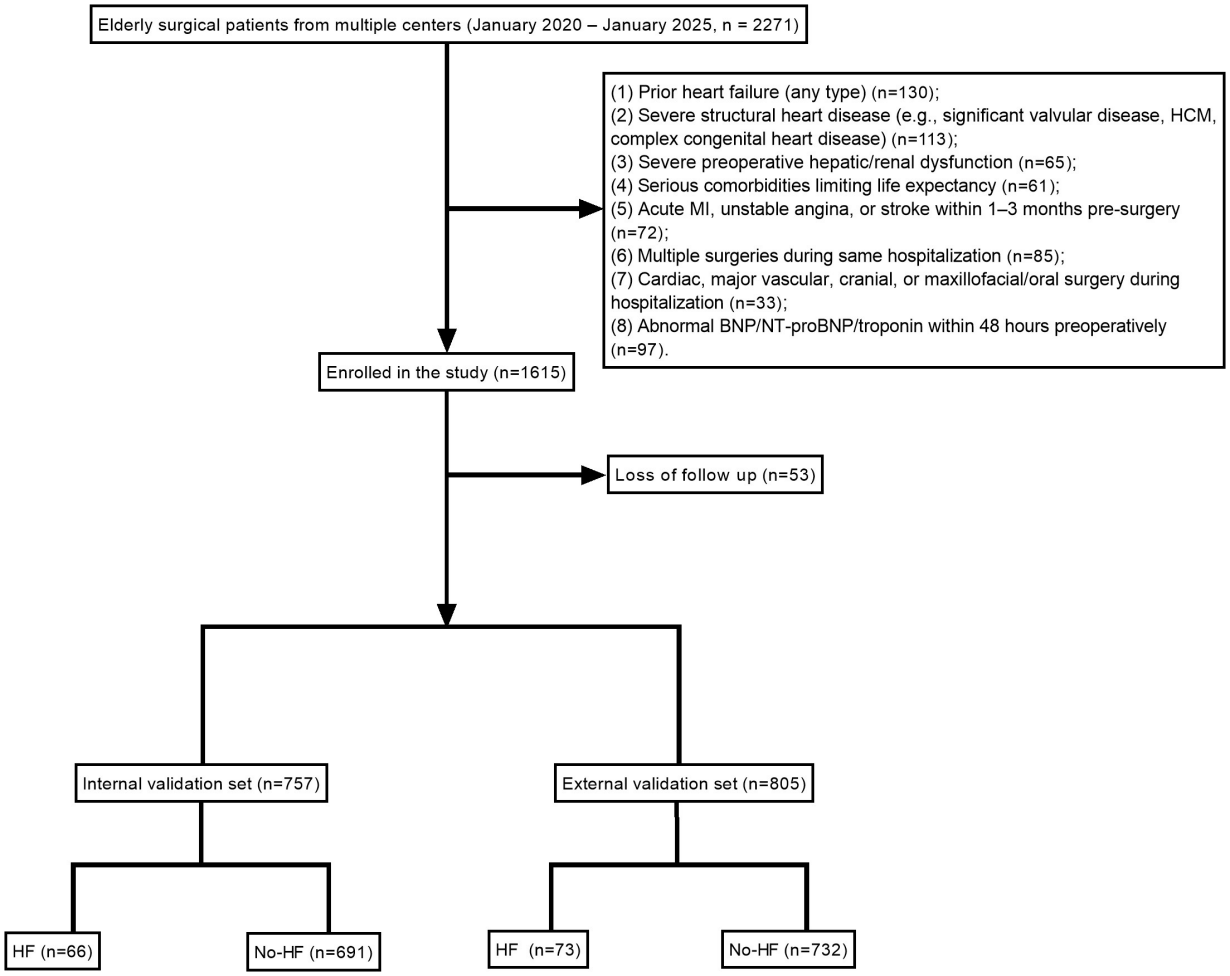
Flow diagram of patient selection based on predefined inclusion and exclusion criteria. The diagram illustrates the stepwise screening process of the study population, including initial patient identification, application of inclusion and exclusion criteria, and final enrollment in the internal and external cohorts.

A total of 1,562 elderly patients were included in this study, with 757 patients assigned to the internal dataset and 805 patients to the external validation dataset (Table 1). Within the internal cohort, 66 patients developed postoperative HF, yielding an incidence of 8.72%. Univariate analysis demonstrated that, compared with the non-HF group, patients in the HF group exhibited significant differences across multiple clinical characteristics. Multivariate analysis further identified extreme age, sex, BMI, hypoalbuminaemia, smoking history, diabetes, hypothyroidism, hypertension, hyperlipidaemia, COPD, malignancy, intraoperative tachycardia, intraoperative hypoxemia, and postoperative NLR, SAA, and CRP levels as independent predictors of postoperative HF (*P* < 0.05) (Figure 1, Table 2).

**Table 1:** Baseline clinical characteristics in the dataset.

Variables		All (*n* = 1,562)	Non-HF (*n* = 1,423)	HF (*n* = 139)	*P*-value
Sex	Female	825 (52.817)	745 (52.354)	80 (57.554)	0.241
	Male	737 (47.183)	678 (47.646)	59 (42.446)	
Age	< 80	790 (50.576)	707 (49.684)	83 (59.712)	0.024
	≥80	772 (49.424)	716 (50.316)	56 (40.288)	
BMI	< 25 kg/m^2^	1,193 (76.376)	1,108 (77.864)	85 (61.151)	< 0.001
	≥25 kg/m^2^	369 (23.624)	315 (22.136)	54 (38.849)	
ASA	< 3	1,125 (72.023)	1,022 (71.820)	103 (74.101)	0.568
	≥3	437 (27.977)	401 (28.180)	36 (25.899)	
ALB	≥30g/L	1,125 (72.023)	1,032 (72.523)	93 (66.906)	0.159
	< 30g/L	437 (27.977)	391 (27.477)	46 (33.094)	
NRS2002 score	< 3	1,158 (74.136)	1,055 (74.139)	103 (74.101)	0.992
	≥3	404 (25.864)	368 (25.861)	36 (25.899)	
Drinking history	No	1,125 (72.023)	1,019 (71.609)	106 (76.259)	0.244
	Yes	437 (27.977)	404 (28.391)	33 (23.741)	
Smoking history	No	1,140 (72.983)	1,038 (72.944)	102 (73.381)	0.912
	Yes	422 (27.017)	385 (27.056)	37 (26.619)	
Surgical history	No	1,279 (81.882)	1,169 (82.150)	110 (79.137)	0.379
	Yes	283 (18.118)	254 (17.850)	29 (20.863)	
Anemia	No	1,408 (90.141)	1,283 (90.162)	125 (89.928)	0.93
	Yes	154 (9.859)	140 (9.838)	14 (10.072)	
Hypothyroidism	No	1,226 (78.489)	1,126 (79.129)	100 (71.942)	0.049
	Yes	336 (21.511)	297 (20.871)	39 (28.058)	
Hyperlipidemia	No	1,174 (75.160)	1,100 (77.301)	74 (53.237)	< 0.001
	Yes	388 (24.840)	323 (22.699)	65 (46.763)	
Hypertension	No	1,242 (79.513)	1,175 (82.572)	67 (48.201)	< 0.001
	Yes	320 (20.487)	248 (17.428)	72 (51.799)	
Diabetes	No	1,259 (80.602)	1,189 (83.556)	70 (50.360)	< 0.001
	Yes	303 (19.398)	234 (16.444)	69 (49.640)	
COPD	No	1,414 (90.525)	1,285 (90.302)	129 (92.806)	0.336
	Yes	148 (9.475)	138 (9.698)	10 (7.194)	
Tumor	No	1,105 (70.743)	1,035 (72.734)	70 (50.360)	< 0.001
	Yes	457 (29.257)	388 (27.266)	69 (49.640)	
Anesthesia type	General anesthesia	1,463 (93.662)	1,341 (94.238)	122 (87.770)	0.003
	Regional anesthesia	99 (6.338)	82 (5.762)	17 (12.230)	
Surgical procedure	Open surgery	1,035 (66.261)	944 (66.339)	91 (65.468)	0.836
	Laparoscopic surgery	527 (33.739)	479 (33.661)	48 (34.532)	
Emergency surgery	No	1,212 (77.593)	1,112 (78.145)	100 (71.942)	0.094
	Yes	350 (22.407)	311 (21.855)	39 (28.058)	
Surgery time	< 270 min	1,102 (70.551)	1,005 (70.625)	97 (69.784)	0.835
	≥270 min	460 (29.449)	418 (29.375)	42 (30.216)	
Intraoperative bleeding	< 100 ml	1,083 (69.334)	983 (69.079)	100 (71.942)	0.485
	≥100 ml	479 (30.666)	440 (30.921)	39 (28.058)	
Intraoperative hypotension	No	965 (61.780)	883 (62.052)	82 (58.993)	0.479
	Yes	597 (38.220)	540 (37.948)	57 (41.007)	
Intraoperative tachycardia	No	1,188 (76.056)	1,123 (78.918)	65 (46.763)	< 0.001
	Yes	374 (23.944)	300 (21.082)	74 (53.237)	
Intraoperative SpO_2_	≥90 %	1,413 (90.461)	1,286 (90.372)	127 (91.367)	0.703
	< 90 %	149 (9.539)	137 (9.628)	12 (8.633)	
Blood transfusion	No	1,408 (90.141)	1,286 (90.372)	122 (87.770)	0.326
	Yes	154 (9.859)	137 (9.628)	17 (12.230)	
Postoperative hypotension	No	1,181 (75.608)	1,066 (74.912)	115 (82.734)	0.04
	Yes	381 (24.392)	357 (25.088)	24 (17.266)	
Postoperative infection	No	1,315 (84.187)	1,198 (84.188)	117 (84.173)	0.996
	Yes	247 (15.813)	225 (15.812)	22 (15.827)	
PCT level	< 0.05 ng/ml	1,275 (81.626)	1,165 (81.869)	110 (79.137)	0.427
	≥0.05 ng/ml	287 (18.374)	258 (18.131)	29 (20.863)	
CRP level	< 10 mg/l	1,181 (75.608)	1,103 (77.512)	78 (56.115)	< 0.001
	≥10 mg/l	381 (24.392)	320 (22.488)	61 (43.885)	
SAA level	< 10 mg/l	1,244 (79.641)	1,141 (80.183)	103 (74.101)	0.089
	≥10 mg/l	318 (20.359)	282 (19.817)	36 (25.899)	
NLR	< 3	1,214 (77.721)	1,134 (79.691)	80 (57.554)	< 0.001
	≥3	348 (22.279)	289 (20.309)	59 (42.446)	
SCr	< 1.2 mg/dl	1,331 (85.211)	1,222 (85.875)	109 (78.417)	0.018
	≥1.2 mg/dl	231 (14.789)	201 (14.125)	30 (21.583)	
BUN	< 20 mg/dl	1,378 (88.220)	1,257 (88.335)	121 (87.050)	0.654
	≥20 mg/dl	184 (11.780)	166 (11.665)	18 (12.950)	
Proteinuria	No	1,396 (89.373)	1,273 (89.459)	123 (88.489)	0.723
	Yes	166 (10.627)	150 (10.541)	16 (11.511)	
Hematuria	No	1,381 (88.412)	1,260 (88.545)	121 (87.050)	0.599
	Yes	181 (11.588)	163 (11.455)	18 (12.950)	

OR, odds ratio; CI, confidence interval; BMI, body mass index; ASA, The American Society of Anesthesiologists; ALB, albumin; PCT, procalcitonin; CRP, C-reactive protein; SAA, serum amyloid A; NRS2002, nutrition risk screening 2002; COPD, chronic obstructive pulmonary disease; SPO_2_, percutaneous arterial oxygen saturation.

**Table 2:** Univariate and multivariate logistic regression analysis for postoperative heart failure.

Variables		Univariate analysis		Multivariate analysis	
		OR, 95%CI	*P*-value	OR, 95%CI	*P*-value
Sex	Female	Reference		Reference	
	Male	0.288 [0.171, 0.484]	0	0.323 [0.178, 0.578]	0
Age	< 80	Reference		–	–
	≥80	0.63 [0.367, 1.081]	0.093		
BMI	< 25 kg/m^2^	Reference		Reference	
	≥25 kg/m^2^	2.032 [1.186, 3.482]	0.01	2.551 [1.355, 4.759]	0.003
ASA	< 3	Reference			
	≥3	1.253 [0.746, 2.104]	0.393		
ALB	≥30g/L	Reference		–	–
	< 30g/L	1.176 [0.701, 1.973]	0.54		
NRS2002 score	< 3	Reference		–	–
	≥3	0.943 [0.545, 1.633]	0.835		
Drinking history	No	Reference		–	–
	Yes	0.883 [0.506, 1.541]	0.661		
Smoking history	No	Reference		–	–
	Yes	0.678 [0.390, 1.181]	0.17		
Surgical history	No	Reference		–	–
	Yes	1.603 [0.903, 2.846]	0.107		
Anemia	No	Reference		–	–
	Yes	1.224 [0.561, 2.668]	0.612		
Hypothyroidism	No	Reference		Reference	
	Yes	1.847 [1.110, 3.072]	0.018	1.710 [0.951, 3.067]	0.071
Hyperlipidemia	No	Reference		Reference	
	Yes	2.329 [1.391, 3.900]	0.001	2.063 [1.129, 3.749]	0.018
Hypertension	No	Reference		Reference	
	Yes	2.613 [1.567, 4.357]	0	1.827 [1.013, 3.273]	0.043
Diabetes	No	Reference		Reference	
	Yes	3.88 [2.316, 6.499]	0	2.363 [1.313, 4.257]	0.004
COPD	No	Reference		–	–
	Yes	0.501 [0.153, 1.643]	0.254		
Tumor	No	Reference		Reference	
	Yes	2.714 [1.624, 4.536]	0	2.386 [1.318, 4.308]	0.004
Anesthesia type	General anesthesia	Reference		–	–
	Regional anesthesia	1.89 [0.852, 4.191]	0.117		
Surgical procedure	Open surgery	Reference		–	–
	Laparoscopic surgery	1.281 [0.688, 2.386]	0.435		
Emergency surgery	No	Reference		–	–
	Yes	1.325 [0.768, 2.285]	0.312		
Surgery time	< 270 min	Reference		–	–
	≥270 min	1.463 [0.853, 2.510]	0.167		
Intraoperative bleeding	< 100 ml	Reference		–	–
	≥100 ml	0.913 [0.523, 1.594]	0.75		
Intraoperative hypotension	No	Reference		–	–
	Yes	1.659 [0.966, 2.852]	0.067		
Intraoperative tachycardia	No	Reference		Reference	
	Yes	4.571 [2.710, 7.710]	0	3.584 [2.010, 6.464]	0
Intraoperative SpO_2_	≥90 %	Reference		–	–
	< 90 %	1.084 [0.499, 2.355]	0.839		
Blood transfusion	No	Reference		–	–
	Yes	1.425 [0.650, 3.122]	0.377		
Postoperative hypotension	No	Reference		–	–
	Yes	0.81 [0.445, 1.477]	0.492		
Postoperative infection	No	Reference		–	–
	Yes	1.111 [0.588, 2.100]	0.747		
PCT level	< 0.05 ng/ml	Reference		–	–
	≥0.05 ng/ml	1.089 [0.586, 2.022]	0.788		
CRP level	< 10 mg/l	Reference		Reference	
	≥10 mg/l	2.515 [1.504, 4.205]	0	2.106 [1.167, 3.785]	0.013
SAA level	< 10 mg/l	Reference		–	–
	≥10 mg/l	0.56 [0.271, 1.157]	0.117		
NLR	< 3	Reference		Reference	
	≥3	3.113 [1.864, 5.199]	0	2.039 [1.128, 3.667]	0.017
SCr	< 1.2 mg/dl	Reference		–	–
	≥1.2 mg/dl	1.318 [0.728, 2.386]	0.362		
BUN	< 20 mg/dl	Reference		–	–
	≥20 mg/dl	1.21 [0.612, 2.393]	0.583		
Proteinuria	No	Reference		–	–
	Yes	0.799 [0.385, 1.660]	0.548		
Hematuria	No	Reference		–	–
	Yes	1.216 [0.642, 2.303]	0.549		

OR, odds ratio; CI, confidence interval; BMI, body mass index; ASA, The American Society of Anesthesiologists; ALB, albumin; PCT, procalcitonin; CRP, C-reactive protein; SAA, serum amyloid A; NRS2002, nutrition risk screening 2002; COPD, chronic obstructive pulmonary disease; SPO_2_, percutaneous arterial oxygen saturation.

**Figure 2:**
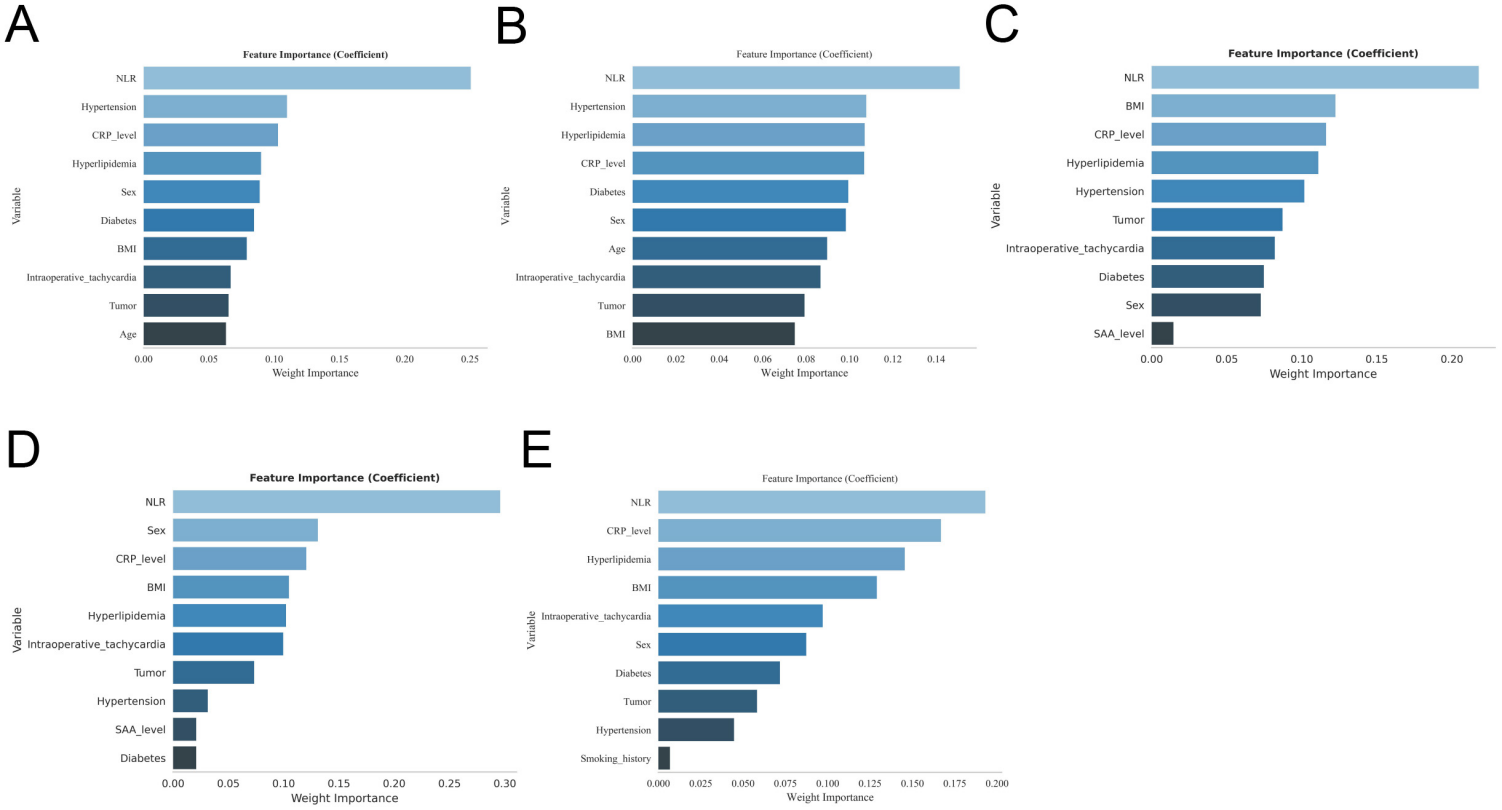
Feature selection results obtained from five machine learning models. **(A)** XGBoost, **(B)** random forest, **(C)** support vector machine, **(D)** k-nearest neighbors, and **(E)** multilayer perceptron. Each panel displays the relative importance or contribution of candidate variables, demonstrating differences in feature selection mechanisms across models.

In machine learning analyses, XGBoost, RF, SVM, MLP, and KNN consistently identified key predictive features, including sex, BMI, history of diabetes, hypertension, hyperlipidaemia, malignancy, intraoperative tachycardia, and postoperative NLR and CRP levels (Figures 2A–E). By integrating traditional statistical analyses with machine learning–derived feature importance, the final predictive model was constructed using nine core variables: sex, BMI, history of diabetes, hypertension, hyperlipidaemia, malignancy, intraoperative tachycardia, and postoperative NLR and CRP levels. The original dataset utilized in this study is provided in Supplementary Table S1.

### Model building and evaluation

**Figure 3:**
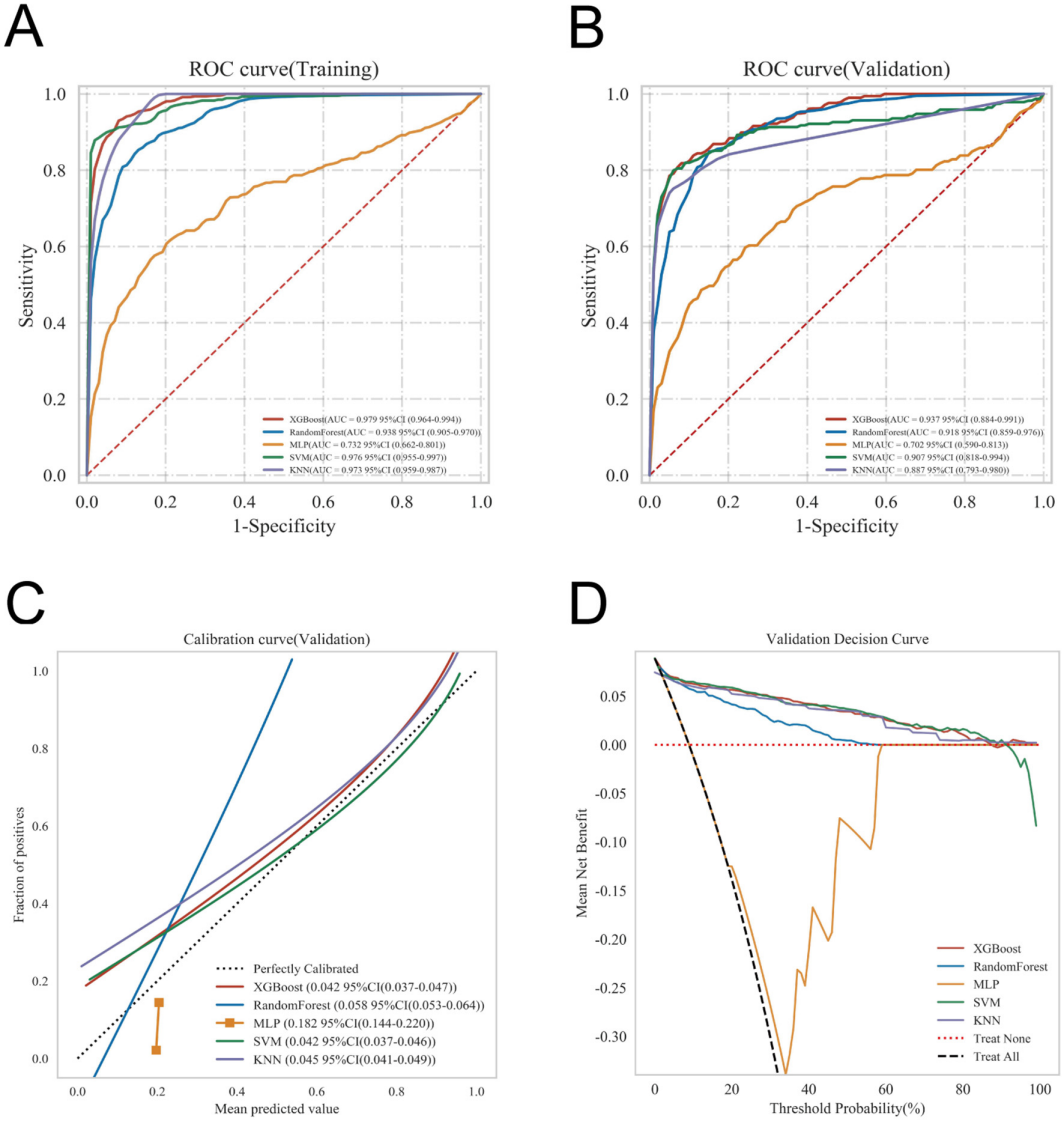
Comparative performance of five machine learning models in predicting postoperative heart failure. **(A)** Receiver operating characteristic (ROC) curves in the training set. **(B)** ROC curves in the validation set. **(C)** Calibration curves assessing agreement between predicted and observed probabilities. **(D)** Decision curve analysis evaluating the clinical net benefit across a range of threshold probabilities.

The ROC curve analysis demonstrated that XGBoost achieved an AUC of 0.979 in the training set and 0.937 in the internal validation set, outperforming the other four models (Table 3, Figures 3A, B). Calibration performance assessment revealed that the calibration curves for XGBoost, SVM, MLP, and KNN closely aligned with the ideal reference line, indicating strong agreement between predicted probabilities and observed outcomes and reflecting reliable predictive accuracy. In contrast, the RF model exhibited some deviation from the ideal line in certain probability ranges, suggesting relatively weaker calibration in estimating risk probabilities (Figure 3C). DCA indicated that XGBoost, SVM, RF, and KNN achieved net clinical benefits across a wide range of threshold probabilities, surpassing the “treat-all” and “treat-none” strategies, thereby demonstrating potential clinical utility. Conversely, the MLP model provided lower net benefit across most threshold ranges, with its curve approaching or falling below the baseline strategies, indicating limited potential for clinical decision support (Figure 3D). In this study, hyperparameters were optimized using a grid search strategy. The key hyperparameter configurations for each model were as follows: for the XGBoost model, the optimal parameters were: colsample_bytree (feature subsampling rate) = 1, learning_rate = 0.1, max_depth = 4, min_child_weight = 2, n_estimators = 5, reg_lambda = 0.5, and subsample = 1. For the Random Forest model (AUC = 0.742), the selected parameters included: criterion = gini, max_depth = None, max_features = sqrt, min_impurity_decrease = 0.0, min_samples_leaf = 1, min_samples_split = 2, and n_estimators = 100. For the MLP model, the optimal configuration was: activation = logistic, alpha = 0.0001, batch_size = auto, hidden_layer_sizes = (10, 10), learning_rate = constant, learning_rate_init = 0.001, max_iter = 10, solver = adam, and tol = 0.0001. For the SVM model, the parameters were: C = 0.1, gamma = scale, kernel = rbf, max_iter = 50, probability = True, and tol = 0.001. For the KNN model (AUC = 0.581), the optimal parameters included: algorithm = auto, leaf_size = 50, *n*_neighbors = 2, *p* = 2, and weights = uniform.

**Table 3:** Comparison of selected features across five machine learning models.

Models		AUC (95%CI)	Accuracy (95%CI)	Sensitivity (95%CI)	Specificity (95%CI)
KNN	Training set	0.973 (0.959–0.987)	0.848(0.828–0.868)	0.988(0.964–1.012)	0.835(0.811–0.859)
	Validation set	0.887 (0.793–0.980)	0.827(0.815–0.839)	0.833(0.790–0.877)	0.827(0.810–0.843)
XGBoost	Training set	0.979 (0.964–0.994)	0.93(0.915–0.945)	0.937(0.922–0.951)	0.929(0.912–0.947)
	Validation set	0.937 (0.884–0.991)	0.907(0.890–0.923)	0.799(0.732–0.865)	0.917(0.895–0.940)
RF	Training set	0.938 (0.905–0.970)	0.879(0.862–0.896)	0.865(0.831–0.899)	0.88(0.859–0.901)
	Validation set	0.918 (0.859–0.976)	0.857(0.826–0.889)	0.788(0.731–0.846)	0.865(0.829–0.901)
SVM	Training set	0.976 (0.955–0.997)	0.968(0.960–0.977)	0.897(0.879–0.915)	0.975(0.965–0.985)
	Validation set	0.907 (0.818–0.994)	0.914(0.895–0.933)	0.805(0.764–0.847)	0.925(0.901–0.949)
MLP	Training set	0.732 (0.662–0.801)	0.801(0.719–0.883)	0.643(0.478–0.809)	0.816(0.721–0.910)
	Validation set	0.702 (0.590–0.813)	0.788(0.708–0.869)	0.571(0.398–0.744)	0.81(0.715–0.904)

CI, confidence interval; KNN, k-nearest neighbor; XGBoost, extreme gradient boosting; RF, random forest; SVM, support vector machine; MLP, multilayer perceptron; AUC, area under the curve.

**Figure 4:**
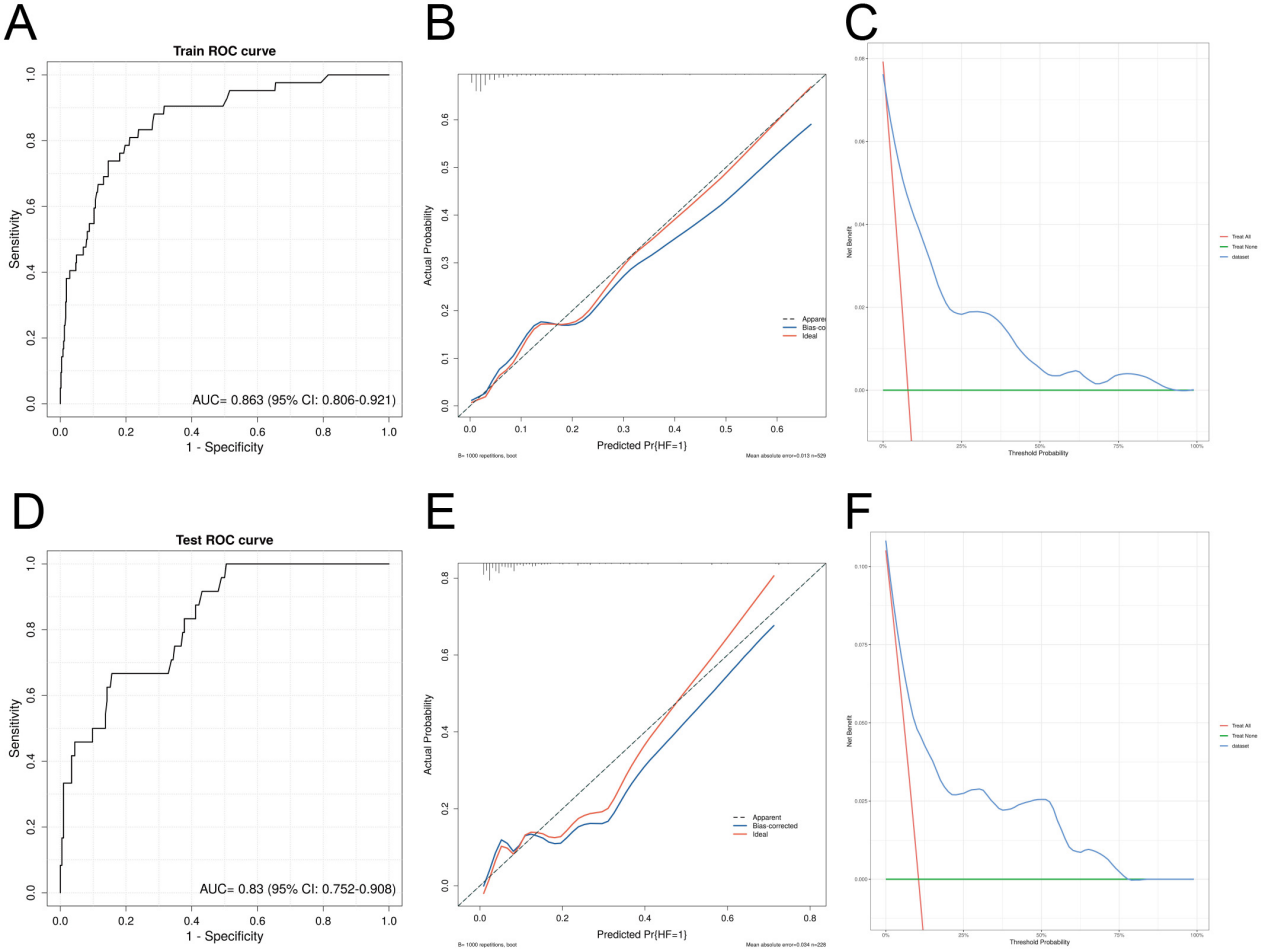
Validation of the logistic regression model in the internal dataset. **(A)** ROC curve in the training set; **(B)** calibration curve in the training set; **(C)** decision curve analysis (DCA) curve in the training set; **(D)** ROC curve in the validation set; **(E)** calibration curve in the validation set; **(F)** DCA curve in the validation set.

In contrast, the traditional logistic regression model using the same clinical indicators achieved AUC values of 0.863 (95% CI: 0.806–0.921) in the training set and 0.830 (95% CI: 0.752–0.908) in the validation set, which were significantly lower than those of the XGBoost model. Although the calibration curve and DCA curve of the logistic regression model showed acceptable performance, its overall predictive performance remained inferior to that of the XGBoost model proposed in this study (Figures 4A–F).

**Figure 5:**
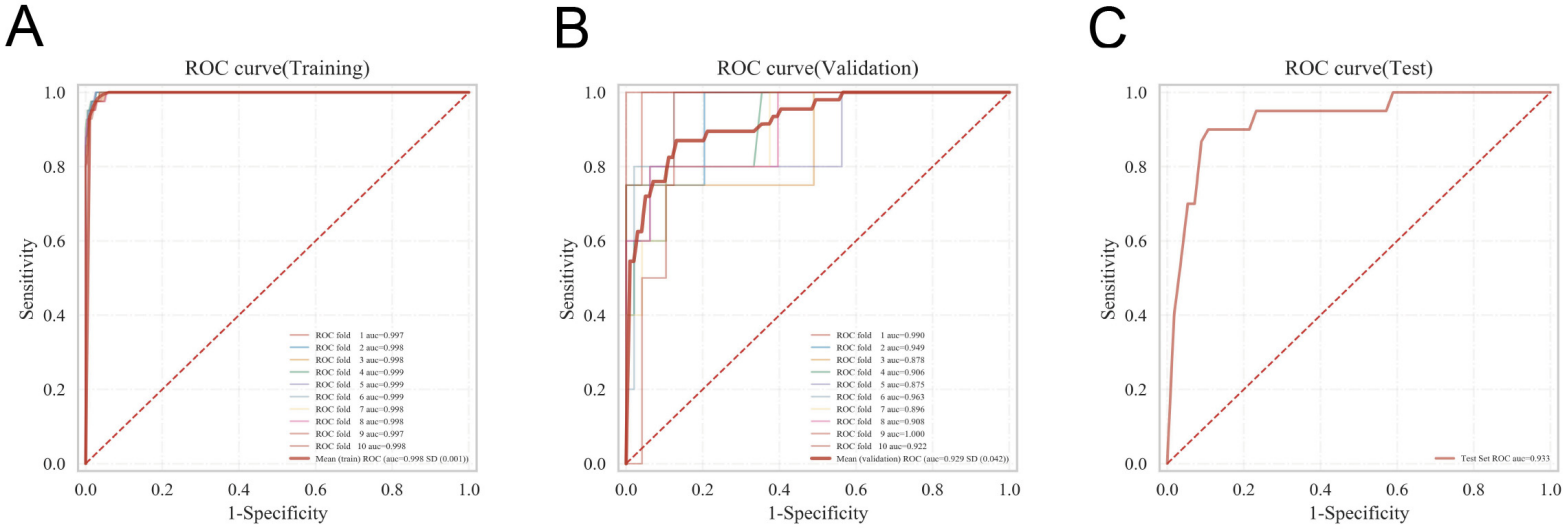
Assessment of the generalization ability of the XGBoost model using k-fold cross-validation. **(A)** ROC curve in the training set. **(B)** ROC curve in the validation set. **(C)** ROC curve in the test set. These results demonstrate the robustness and stability of the model across different data partitions.

To compare generalizability among the five models, tenfold cross-validation was performed. From the internal validation cohort, 228 patients (30.12%) were randomly selected as the validation set, with the remaining patients used as the training set. Results showed that XGBoost achieved an AUC of 0.9286 ± 0.0423 in the validation folds, with a test set AUC of 0.9327 and an accuracy of 0.9079 (Figures 5A–C). RF yielded a validation AUC of 0.9231 ± 0.0442, test set AUC of 0.9304, and accuracy of 0.9035; SVM achieved a validation AUC of 0.9203 ± 0.0618, test set AUC of 0.9318, and accuracy of 0.9298; KNN had a validation AUC of 0.8451 ± 0.0790, test set AUC of 0.8500, and accuracy of 0.8991; and MLP achieved a validation AUC of 0.9263 ± 0.0468, test set AUC of 0.9180, and accuracy of 0.9386. Based on these comparisons, XGBoost was selected for final model construction.

**Figure 6:**
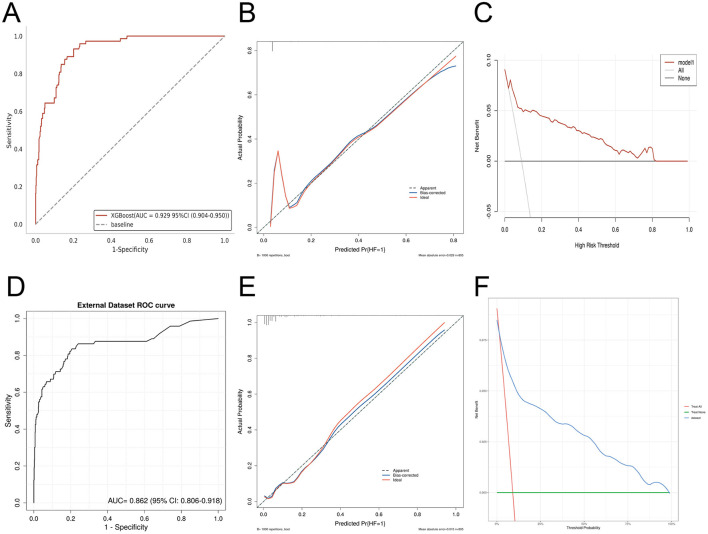
External validation of the XGBoost model and the traditional logistic regression model. **(A)** ROC curve of the XGBoost model evaluating discriminative performance. **(B)** Calibration curve of the XGBoost model assessing predictive accuracy. **(C)** Decision curve analysis (DCA) of the XGBoost model illustrating clinical utility. **(D)** ROC curve of the traditional logistic regression model. **(E)** Calibration curve of the traditional logistic regression model. **(F)** DCA curve of the traditional logistic regression model.

**Figure 7:**
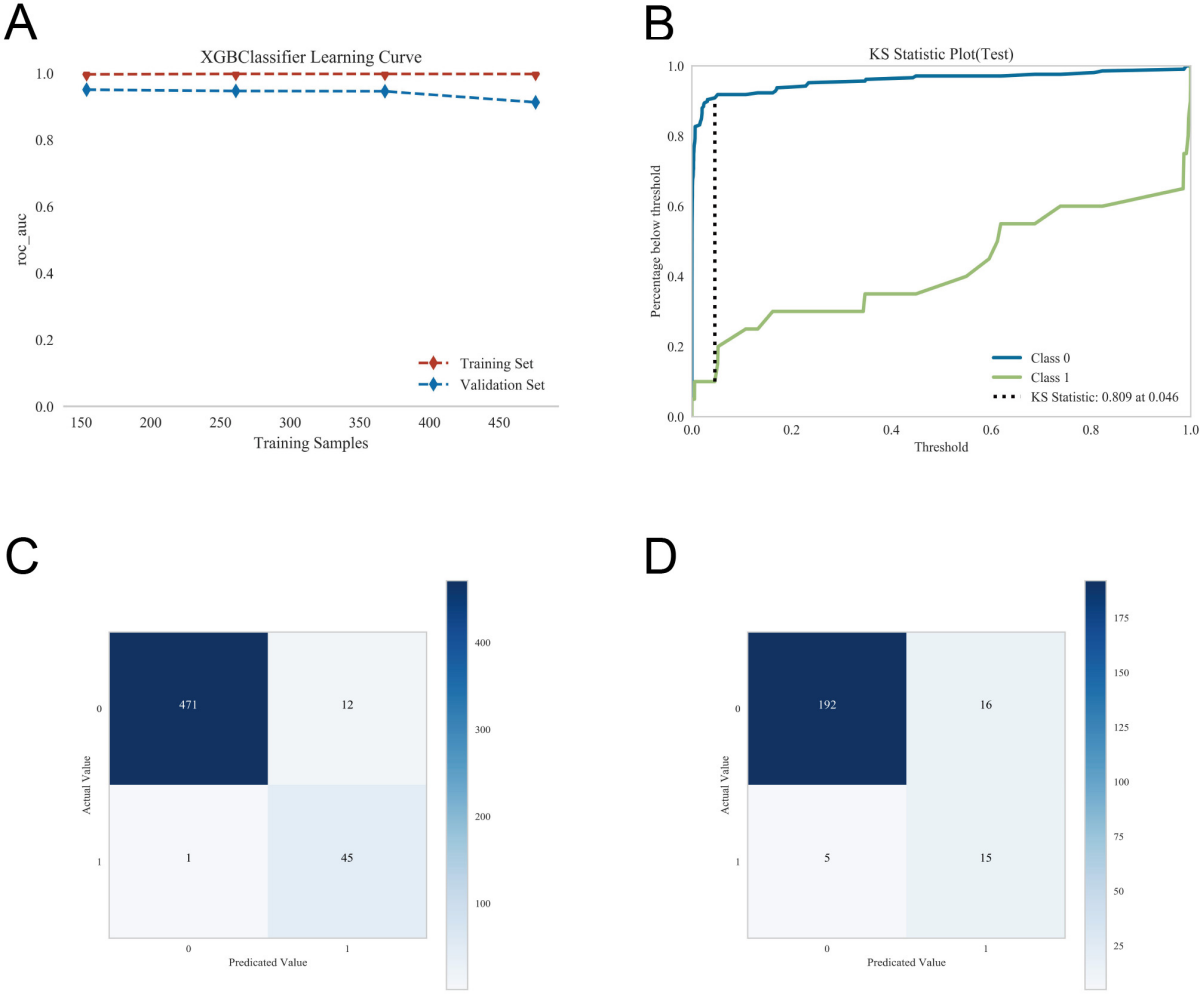
Further evaluation of the performance and stability of the XGBoost model. **(A)** Learning curve showing model performance as a function of training sample size. **(B)** Kolmogorov–Smirnov (KS) curve evaluating the model's discriminatory ability. **(C)** Confusion matrix in the training set. **(D)** Confusion matrix in the test set. These analyses provide additional insight into model fitting, discrimination, and classification performance.

In the external validation cohort, ROC analysis confirmed robust discriminative ability, with an AUC of 0.92. Calibration curves and DCA further demonstrated satisfactory agreement between predicted and observed outcomes and highlighted potential clinical utility (Figures 6A–C). In this study, the traditional logistic regression model was also used to validate the external dataset, achieving an AUC of 0.862 (95% CI: 0.806–0.918), which was inferior to that of the XGBoost model. The DCA curve and calibration curve showed acceptable performance (Figures 6D–F). Additional performance evaluations indicated that the model exhibited strong discrimination, as evidenced by KS analysis. Learning curves demonstrated stabilization of performance with increasing training sample size, without obvious overfitting or underfitting. Confusion matrices for both the training and validation sets showed high classification accuracy and consistent predictive performance (Figures 7A–D).

### Model interpretability via SHAP analysis

**Figure 8:**
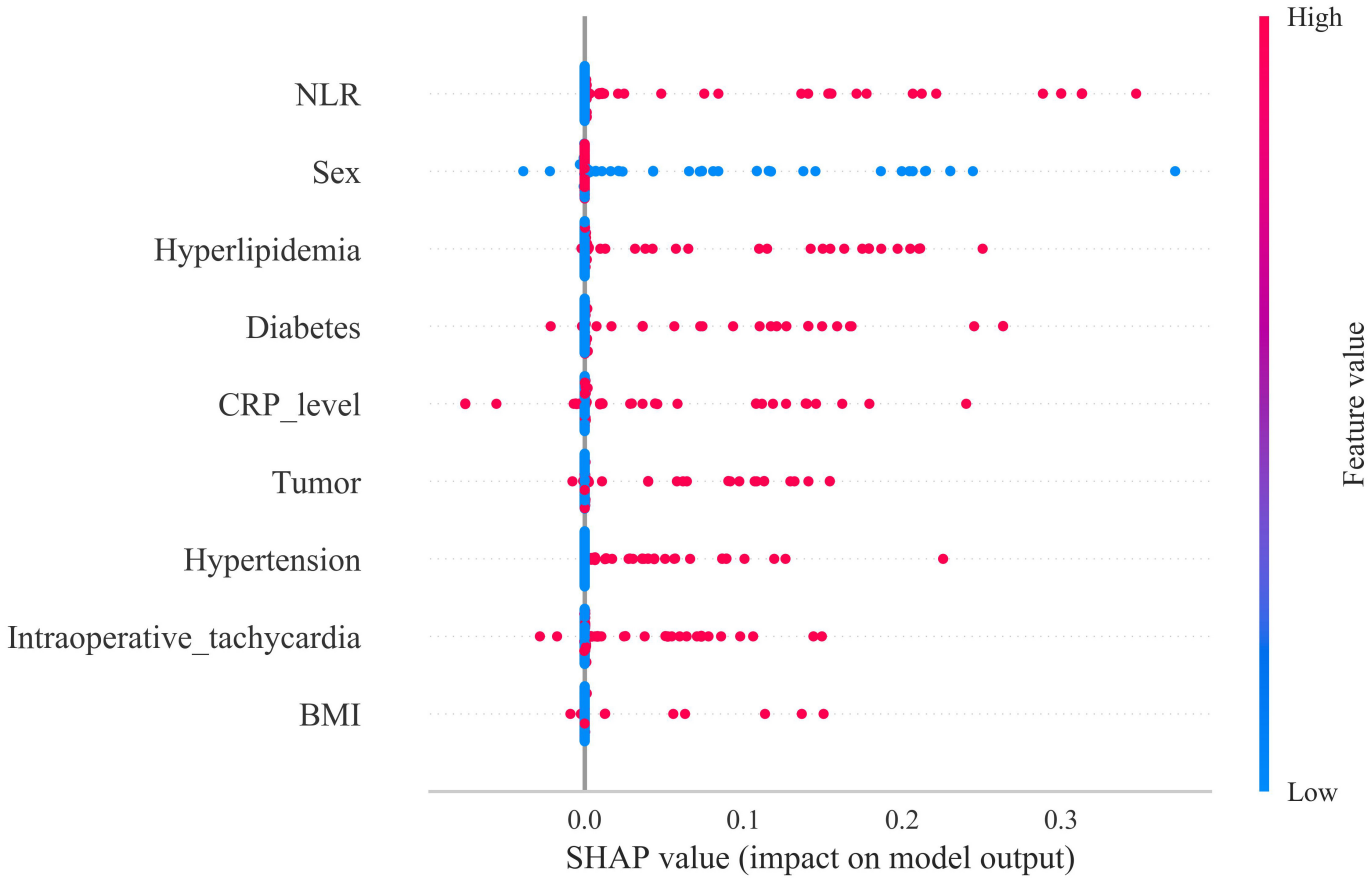
Global interpretability of the XGBoost model using SHAP analysis. The SHAP summary plot illustrates the overall contribution and importance of each feature, as well as the direction and magnitude of their effects on model predictions.

The predictive model was further subjected to interpretative evaluation using SHAP to quantify the contribution of each variable to the risk of postoperative heart failure. The SHAP summary plot ranked feature importance from highest to lowest as follows: postoperative NLR, female sex, hyperlipidaemia, diabetes mellitus, postoperative CRP, history of malignancy, hypertension, intraoperative tachycardia, and elevated BMI. Among these, postoperative NLR and CRP—representing systemic inflammatory markers—exerted the greatest influence on HF risk, highlighting the pivotal role of systemic inflammation in postoperative cardiac dysfunction. Baseline metabolic conditions such as hyperlipidaemia, diabetes, and hypertension also markedly increased risk, reflecting the chronic metabolic burden on cardiac functional reserve. Additionally, perioperative tachycardia, female sex, and elevated BMI contributed to heightened risk states to varying degrees (Figure 8).

**Figure 9:**
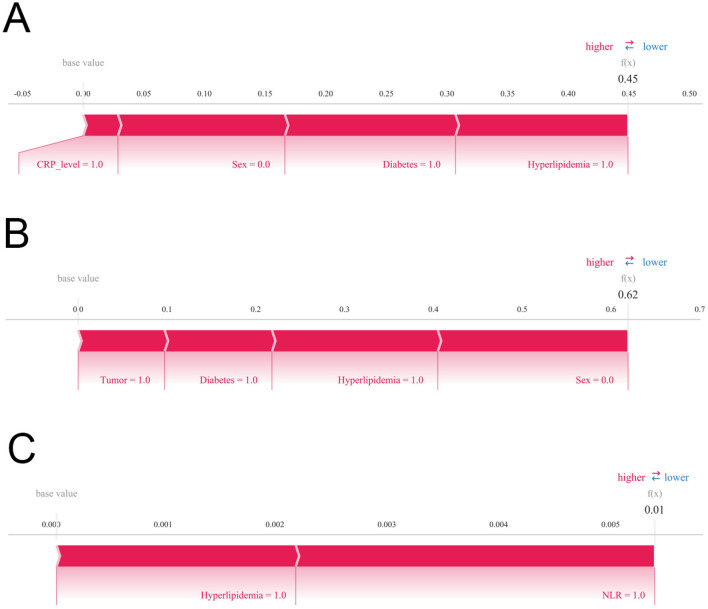
Individual-level interpretability of the XGBoost model using SHAP force plots. **(A–C)** Representative examples from three individual patients. Each force plot visualizes how specific features contribute to the predicted risk of postoperative heart failure at the individual level.

Moreover, individualized SHAP decision plots were generated for three patients who developed postoperative HF to elucidate key drivers of risk prediction at the patient level. Patient 1 had a predicted probability of 0.45, indicating a moderate-to-high risk. The SHAP force plot demonstrated that several high-risk features pushed the prediction toward HF, with the largest Shapley values corresponding to elevated postoperative CRP, female sex, history of diabetes, and hyperlipidaemia, suggesting these factors were principal contributors to the high-risk classification. Patient 2, with a predicted probability of 0.62, represented a relatively high-risk individual. The SHAP force plot indicated that malignancy history, female sex, diabetes, and hyperlipidaemia significantly increased the predicted probability, confirming their critical role in maintaining a high-risk status. In contrast, Patient 3 had a predicted probability of 0.01, representing a low-risk profile. The force plot showed that minor elevations in postoperative NLR and hyperlipidaemia contributed modestly, but their combined effect was insufficient to substantially increase HF risk, resulting in a low-risk classification by the model (Figures 9A–C).

## Discussion

In this study, we systematically evaluated the predictive performance of five machine learning algorithms—XGBoost, RF, SVM, MLP, and KNN—for forecasting postoperative HF in elderly patients. XGBoost emerged as the superior model, demonstrating the highest discriminative power and stability across both training and validation cohorts, while retaining robust performance in an external validation cohort. Its calibration and decision curve analyses further underscored its substantial clinical utility. This exceptional performance likely reflects XGBoost's iterative optimization, enabling it to capture complex nonlinear interactions among variables with high fidelity, while simultaneously offering strong resistance to overfitting and interpretability (28–30). By contrast, RF, although stable in classification, exhibited deviations in calibration across certain probability ranges, indicative of slightly diminished risk estimation accuracy (31). SVM, adept at handling nonlinearly separable data, is constrained by high computational demands and reduced efficiency on large datasets. MLP accommodates intricate nonlinear relationships but is prone to overfitting and sensitive to sample size, yielding relatively modest clinical net benefit. KNN, while conceptually simple and intuitive, relies heavily on sample distribution and is susceptible to noise (32). Considering discriminative performance, calibration, clinical utility, and generalizability, we ultimately selected XGBoost to construct the postoperative HF predictive model, offering a robust tool for early risk stratification and individualized perioperative management in elderly patients.

Building upon this high-performance model, we probed the specific determinants of postoperative HF and their underlying mechanisms, with the aim of translating predictive insights into actionable clinical interventions. SHAP analysis identified female sex as an independent risk factor for postoperative HF in elderly patients, likely reflecting sex-specific differences in cardiac architecture, hormonal milieu, and other physiological parameters. Compared with males, females generally exhibit smaller left ventricular cavities, thicker ventricular walls, and reduced myocardial compliance (33, 34), traits that are accentuated with advancing age. During perioperative volume shifts, fluid resuscitation, or hemodynamic perturbations, this relatively diminished diastolic reserve predisposes females to elevated left ventricular filling pressures and pulmonary congestion, thereby increasing the likelihood of postoperative HF (35, 36). Furthermore, elderly females are more susceptible to concentric ventricular remodeling, which exacerbates diastolic stiffness and compromises cardiac compensatory capacity under surgical stress. Postmenopausal estrogen decline further attenuates cardioprotective effects, contributing to endothelial dysfunction, reduced nitric oxide (NO) bioavailability, and diminished vascular compliance, collectively augmenting afterload and impairing coronary perfusion. Estrogen's anti-inflammatory and antifibrotic properties are likewise curtailed, promoting myocardial interstitial fibrosis, increased myocardial stiffness, and impaired diastolic function (37, 38). Under the compounded influence of these structural and functional alterations, elderly females are rendered particularly vulnerable to perioperative cardiac decompensation, thereby elevating their risk of postoperative HF.

Furthermore, we observed that elderly patients with concomitant malignancies faced a markedly heightened risk of postoperative HF. This vulnerability likely reflects a convergence of mechanisms, including tumor-associated chronic inflammation, potential cardiotoxic effects of anticancer therapies, and perturbations in metabolic and nutritional status (39–41). Our analyses also revealed that elevated CRP and NLR levels were strongly correlated with postoperative HF, underscoring the pivotal role of systemic inflammation in its pathogenesis. Tumor progression is frequently accompanied by sustained systemic inflammatory responses, with tumor cells and their microenvironment releasing diverse inflammatory mediators, among which IL-6 serves as a central proinflammatory cytokine. IL-6 not only drives acute-phase protein synthesis, elevating CRP levels, but also induces oxidative stress and activates inflammatory signaling pathways, directly injuring cardiomyocytes and promoting myocardial interstitial fibrosis and ventricular remodeling, ultimately impairing systolic and diastolic function (42, 43).

Concurrently, NLR, reflecting an imbalance between neutrophil-driven inflammation and lymphocyte-mediated immune regulation, is elevated in states of heightened inflammatory activity. Persistent inflammation exacerbates endothelial dysfunction, facilitates infiltration of inflammatory cells into myocardial tissue, and triggers cardiomyocyte apoptosis and structural remodeling (44–48). In this study, both CRP and NLR were identified as salient high-risk factors, suggesting that systemic inflammation in cancer patients contributes to myocardial injury and ventricular dysfunction through multiple convergent pathways. Perioperative surgical trauma and physiological stress may further amplify this inflammatory burden, heightening susceptibility to cardiac decompensation.

Moreover, prior exposure to chemotherapy, targeted therapy, or radiotherapy can impose additional cardiotoxic stress. Chemotherapeutic agents may induce myocardial injury via oxidative stress, mitochondrial dysfunction, and apoptosis, while radiotherapy can provoke coronary endothelial damage and myocardial fibrosis. Such treatment-related injuries often evolve insidiously, gradually diminishing cardiac reserve and increasing vulnerability during the perioperative period. Cancer-associated malnutrition, cachexia, and metabolic derangements further compromise cardiac function: chronic energy depletion and protein catabolism reduce skeletal and myocardial mass, impairing contractility, while abnormal fluid distribution imposes additional circulatory strain. In the context of surgical trauma, anesthesia, and perioperative hemodynamic fluctuations, these factors act synergistically to elevate the risk of cardiac decompensation.

In line with prior investigations, hypertension emerged as a salient risk factor for postoperative HF. Chronic elevation of blood pressure exerts persistent structural and functional strain on the heart, markedly diminishing its compensatory capacity under perioperative stress (49, 50). Sustained pressure overload promotes left ventricular hypertrophy, thereby elevating the risk of postoperative HF. Progressive myocardial remodeling, characterized by cardiomyocyte hypertrophy and interstitial collagen deposition, thickens the ventricular wall and reduces compliance, culminating predominantly in diastolic dysfunction (51, 52). These structural alterations are particularly pronounced in elderly patients, rendering the ventricle less capable of maintaining adequate filling and ejection during volume or hemodynamic perturbations.

SHAP analysis further identified diabetes as a potent predictor of postoperative HF. Chronic hyperglycemia in diabetic patients amplifies oxidative stress and fosters the accumulation of advanced glycation end products (AGEs), which directly injure cardiomyocytes and stimulate interstitial collagen deposition, leading to myocardial fibrosis, increased stiffness, and impaired ventricular diastolic function (53–55). Diabetes also predisposes to coronary microvascular disease and endothelial dysfunction, compromising myocardial perfusion and oxygen delivery, and further undermining both systolic and diastolic performance. Perioperative stressors—including anesthesia and surgical trauma—activate sympathetic and inflammatory pathways, elevating catecholamine and cytokine levels, which in turn increase heart rate, blood pressure variability, and myocardial oxygen demand. In the context of preexisting microvascular and metabolic impairments, diabetic patients possess reduced cardiac reserve and are particularly vulnerable to supply-demand mismatch, which is exacerbated by perioperative glycemic fluctuations, oxidative stress, and inflammation, collectively heightening the risk of postoperative HF (56, 57).

Moreover, postoperative HF was intricately linked to metabolic status. Elevated BMI and dyslipidemia, hallmarks of metabolic derangement, were strongly associated with heightened HF risk. The underlying mechanisms likely encompass maladaptive cardiac remodeling, metabolic dysregulation, vascular dysfunction, and amplified perioperative stress responses, collectively augmenting cardiac workload and diminishing compensatory capacity (58, 59). Increased BMI, particularly in overweight or obese individuals, elevates circulating blood volume and cardiac output, precipitating left ventricular hypertrophy and structural remodeling (60, 61). Adipose tissue functions as an active endocrine organ, secreting proinflammatory cytokines such as TNF-α and interleukins, which foster chronic low-grade inflammation and myocardial fibrosis, reduce ventricular compliance, and impair overall cardiac performance. Perioperatively, obese patients frequently contend with elevated oxygen demand, compromised ventilation, and hemodynamic instability, imposing further strain on cardiac function. Dyslipidemia exacerbates atherosclerotic progression and lipid deposition, damaging coronary endothelium and limiting myocardial oxygen delivery; intracellular lipid accumulation, or “lipotoxicity,” provokes cardiomyocyte apoptosis, mitochondrial dysfunction, and fibrotic remodeling, thereby impairing both systolic and diastolic function. Under the compounded stress of surgery and anesthesia, metabolic demands escalate and myocardial oxygen consumption rises, rendering patients with compromised coronary reserve particularly susceptible to ischemia and functional decline.

Perioperative tachycardia emerged as a critical determinant of postoperative HF in elderly patients. Elevated heart rate markedly increases myocardial oxygen demand, and in the context of diminished cardiac reserve characteristic of advanced age, shortened diastolic intervals limit coronary perfusion, precipitating ischemia and impaired contractile function (62). Tachycardia often reflects sympathetic overactivation triggered by anesthesia, surgical stimuli, hemorrhage, hypotension, or pain. Sustained sympathetic drive elevates heart rate, blood pressure variability, and afterload, potentially overwhelming compensatory mechanisms in patients with preexisting cardiac pathology and precipitating decompensation (63). Rapid myocardial contraction further exacerbates metabolic derangements and inflammatory responses, with elevated perioperative IL-6 and CRP compromising myocardial compliance. Collectively, these factors render perioperative tachycardia a potent enhancer of postoperative HF risk.

Compared with established risk prediction tools such as the Revised Cardiac Risk Index (RCRI) and the NSQIP risk calculator, our model demonstrates several distinct advantages and complementary limitations across multiple dimensions. In terms of predictive performance, RCRI is a widely used and well-validated tool for estimating perioperative cardiac risk; however, it relies on a limited number of predefined variables and assumes largely linear relationships, which may constrain its discriminative capacity, particularly in complex and heterogeneous populations such as elderly surgical patients. Similarly, the NSQIP risk calculator incorporates a broader set of clinical variables and has shown good predictive ability across diverse surgical populations, yet its performance may vary depending on procedure type and patient characteristics. In contrast, our XGBoost-based model leverages machine learning to capture complex nonlinear interactions and high-order feature relationships, resulting in superior discrimination, calibration, and clinical net benefit, as demonstrated in both internal and external validation cohorts. Regarding usability, RCRI offers clear advantages due to its simplicity and rapid bedside applicability, requiring only a small number of easily obtainable clinical variables. The NSQIP calculator, although more comprehensive, is typically implemented as an online tool and may require more time for data input. With respect to data requirements, RCRI demands minimal input variables, enhancing its practicality but limiting its granularity. The NSQIP calculator requires a larger set of perioperative variables, including procedural details, which may not always be readily available in all settings. Our model incorporates a more comprehensive set of perioperative variables, including intraoperative and early postoperative parameters, thereby improving predictive accuracy but also necessitating more extensive data collection. This may limit its applicability in resource-constrained environments or in strictly preoperative decision-making scenarios. Taken together, while traditional tools such as RCRI and NSQIP remain valuable for rapid and standardized risk assessment, our machine learning–based model provides enhanced predictive performance and individualized risk stratification by integrating multidimensional clinical data. These approaches should be viewed as complementary, with our model offering particular advantages in settings where detailed perioperative data and digital infrastructure are available.

This study employed a retrospective design and is therefore inherently susceptible to bias, including incomplete medical records and inconsistencies in data acquisition. Although the multicenter design enhances the generalizability of the findings, inter-center variability in perioperative management, diagnostic equipment, and surgical practices may still have influenced the results. Moreover, the validation datasets were largely derived from multiple hospitals within the same healthcare system; while this supports the internal consistency of the model, further external validation across geographically diverse regions and heterogeneous populations is warranted to more rigorously assess its generalizability. Regarding inclusion and exclusion criteria, patients with abnormal preoperative BNP/NT-proBNP/troponin levels were strictly excluded to minimize confounding from pre-existing cardiac dysfunction. While this approach improves cohort homogeneity and strengthens internal validity, it limits the model's ability to predict ultra-early postoperative HF (within 48 h). Furthermore, this criterion constrains the applicability of the findings to patients with pre-existing cardiac impairment, and the model's performance in such populations remains to be established, necessitating validation in broader clinical settings. In terms of feature selection, a multi-strategy cross-validation framework was adopted to identify clinically relevant predictors, which may introduce a degree of selection bias. Although K-fold cross-validation was implemented to mitigate this issue, the possibility of residual optimism bias cannot be entirely excluded. With respect to study objectives, the present work primarily focused on evaluating the predictive performance and interpretability of the model using retrospective multicenter data, and a dedicated web-based application has not yet been developed. In response to this suggestion, we have identified this as a key direction for future research and plan to develop an interactive web-based calculator (e.g., using Streamlit or R Shiny) to facilitate real-time risk estimation by allowing clinicians to input patient data in a user-friendly interface.

In conclusion, we developed and externally validated a postoperative HF prediction model using multicenter data from elderly patients. XGBoost outperformed conventional machine learning approaches in discrimination, calibration, and clinical net benefit. SHAP analysis highlighted sex, diabetes, hypertension, dyslipidemia, elevated BMI, malignancy, perioperative tachycardia, and postoperative inflammatory markers (NLR, CRP) as principal high-risk factors. These findings underscore that perioperative HF is a multifactorial phenomenon, shaped by baseline metabolic disturbances, systemic inflammation, and the structural and functional status of the heart.

## Conclusion

In summary, we developed and validated a robust XGBoost–based predictive model for postoperative HF in elderly patients, surpassing other machine learning approaches in discriminative performance, calibration, and clinical net benefit. SHAP analysis identified sex, diabetes, hypertension, dyslipidemia, elevated BMI, malignancy, perioperative tachycardia, and postoperative inflammatory markers (NLR, CRP) as principal high-risk factors, highlighting the interplay of metabolic derangements, inflammation, and cardiac structural-functional status in perioperative HF pathogenesis.

## Data Availability

The original contributions presented in the study are included in the article/Supplementary material, further inquiries can be directed to the corresponding authors.

## Supplementary material

Supplementary Table S1 Raw Data.
